# Functional Characterization of *VvSR34a* Gene from Grapevine in Response to Salt Stress

**DOI:** 10.3390/plants15071092

**Published:** 2026-04-02

**Authors:** Yu Li, Zhen Gao, Yinping Li, Yuanpeng Du, Haibo Wang

**Affiliations:** 1 College of Horticulture Science and Engineering, Shandong Agricultural University, Taian 271018, China; 2 Research Institute of Pomology, Chinese Academy of Agricultural Sciences, Xingcheng 125100, China

**Keywords:** grape, *VvSR34a* gene, *VvCOP9* gene, salt stress, salt tolerance

## Abstract

Salt stress severely restricts grape (*Vitis vinifera* L.) production. Serine/arginine-rich (SR) proteins, as a class of RNA-binding proteins, play important roles in plant growth, development and stress responses. However, the function and regulatory mechanism of *VvSR34a* in grape salt tolerance remain unclear. In this study, grape callus and cutting seedlings were used as materials to explore the role and molecular mechanism of *VvSR34a* in grape salt stress response. The results showed that, under 100 mM NaCl treatment, the relative level of *VvSR34a* in grape callus exhibited a ‘first increase and then decrease’ pattern, reaching a peak at 2 h, and the gene was localized in the nucleus. Transgenic experiments confirmed that the overexpression of *VvSR34a* significantly enhanced salt tolerance in grape callus and cuttings, as evidenced by better growth status, higher chlorophyll content and root activity, as well as lower electrolyte leakage and malondialdehyde (MDA) content under salt stress. In contrast, the silencing of *VvSR34a* significantly increased salt sensitivity in grapes. Y2H and LCI assays verified that VvSR34a physically interacts with VvCOP9. *VvCOP9* may play a negative regulatory role in the salt stress response of the grapevine, and through the loss of the high salt-tolerant phenotype in the *VvSR34a/VvCOP9*-RNAi lines, it demonstrated that *VvCOP9* is genetically upstream of *VvSR34a*. Furthermore, the ubiquitination and degradation assay demonstrated that VvCOP9 can significantly promote the degradation of VvSR34a. RNA-seq analysis showed that a total of 2834 differentially expressed genes and 202 alternative splicing events were detected in *VvSR34a* overexpression lines. These differentially expressed genes were significantly enriched in ATPase activity, redox and hormone signaling pathways. This study demonstrates that *VvSR34a* positively regulates salt tolerance in grapes, providing an important theoretical basis for molecular breeding of salt-tolerant grapevines.

## 1. Introduction

Grape (*Vitis vinifera* L.) is one of the most widely cultivated fruit crops globally, playing a pivotal role in the global agricultural economy and food industry. Its industrial chain covers table grapes, wine production, and raisin processing, with products meeting daily consumption demands and supporting key sectors such as winemaking and food processing [1]. However, escalating soil salinization driven by global climate change and irrational agricultural irrigation has become a major threat to grape production: High salt levels induce ion toxicity, osmotic stress, membrane damage, reactive oxygen species (ROS) accumulation, metabolic disorders, and inhibited photosynthesis, severely impairing grape growth, development, quality, and yield [2].

Plants have evolved complex regulatory networks to adapt to abiotic stresses, with post-transcriptional and post-translational gene regulation being critical for stress survival [3]. Alternative splicing (AS), a universal and highly dynamic post-transcriptional regulatory process, enables plants to rapidly reprogram gene expression by generating multiple mature mRNA isoforms from a single pre-mRNA, thus adjusting the abundance and function of stress-responsive proteins to cope with environmental stresses [4]. AS patterns change significantly in response to different stresses [5], and stress-resistant genes in *Arabidopsis thaliana*, *Oryza sativa*, *Hordeum vulgare* and *Triticum aestivum* undergo AS under environmental stress, with cell signal transduction-related genes helping plants adapt to drought, high salt, waterlogging and low-temperature stress via AS mode changes [6,7]. AS is regulated by cis-regulatory elements and trans-acting factors, with serine/arginine-rich (SR) proteins—core regulators containing RS domains (rich in S/R repeats) and unique RRM domains—playing key roles in spliceosome assembly and AS modulation [8].

SR proteins have been extensively characterized in model plants and grapes for their roles in stress responses. The related retained research findings are as follows: *SR45* negatively regulates glucose and abscisic acid (ABA) signaling during *Arabidopsis* seed germination. It exerts this regulatory effect by affecting ABA accumulation and the expression of ABA biosynthesis and signaling genes (*ABI3*, *ABI5*). Consistently, the *sr45-1* mutants show high sensitivity to glucose and ABA in early seedling development [9]. Under high-temperature stress, the abundance of *SR45*, *SR30*, *SR34* and U1 snRNP in grapes is significantly increased [10]. In *Arabidopsis*, *AtSR45a* mediates AS and mRNA maturation of salt-tolerant genes, and it participates in post-transcriptional regulation of salt tolerance [11]. The relative level of *Arabidopsis AtSR34b* is induced by cadmium (Cd) stress. The *sr34b* mutant shows moderate Cd sensitivity, accompanied by enhanced Cd accumulation [12]. The overexpression of *OsSCL30* reduces transgenic rice resistance to low-temperature, drought and salt stress. This overexpression also leads to increased ROS accumulation [13]. Loss-of-function mutants of *Arabidopsis* SR family members *rs40*, *rs41* and *scl30a* show hypersensitivity to salt stress [14]. In rice, abiotic stresses (ABA, NaCl, heat, cold) induce AS of SR transcripts. The *rs33* loss-of-function mutants showing hypersensitivity to salt and low-temperature stress. This is because *OsRS33* regulates pre-mRNA splicing in response to abiotic stress [15]. *OsSCR106* is localized to nuclear splicing spots, and regulated pre-mRNA splicing under abiotic stress. The *scr106* loss-of-function mutants show sensitivity to salt, ABA and low-temperature stress. This mutant also exhibits obvious developmental abnormalities [16]. *Arabidopsis* SRRM1L, an SR-related protein with a PWI domain, regulates splice site selection by interacting with U1-70K. Under salt stress, it promotes the splicing of *NFYA10* pre-mRNA. This splicing process generates a functional transcription factor variant that enhances salt tolerance. In contrast, the *srrm1l* mutants retain the first intron of *NFYA10*, producing a non-functional variant and causing salt sensitivity [17].

However, as a member of the SR protein family, SR34a is still unknown in grapes. In this study, we focused on *VvSR34a*, characterized its function in grape salt tolerance via genetic transformation, and further dissected its molecular mechanism in grape salt tolerance. Our research will not only help to perfect the theoretical system of post-transcriptional regulation of salt tolerance in grapes, but also provide brand-new gene targets and theoretical support for molecular breeding of salt tolerance in grapes.

## 2. Results

### 2.1. Expression of VvSR34a Gene in Response to Salt Stress

**Figure 1:**
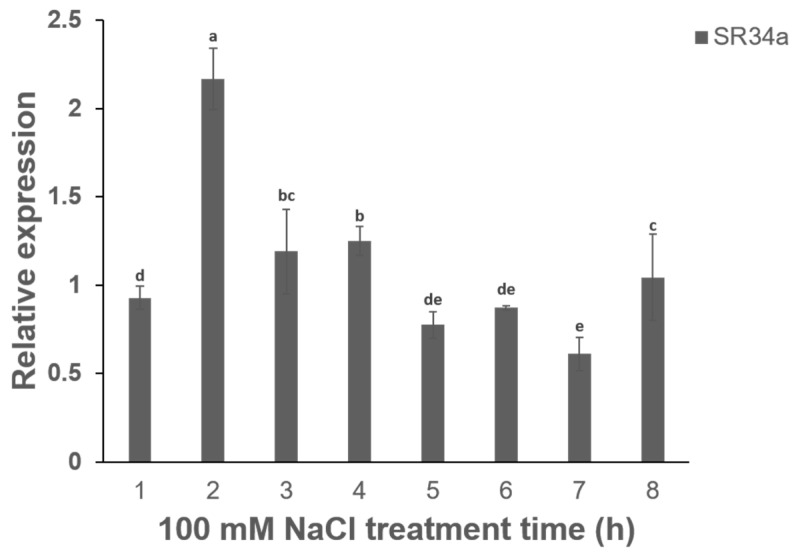
Analysis of relative level of transcripts at different times under 100 mM NaCl treatment. *VvActin* serves as a reference gene. Each value represents the average ± SD of three independent biological replicates. The significant difference between means (*p* < 0.05) is represented by different letters.

In order to explore the potential function of *VvSR34a* under salt stress, we determined the relative level of *VvSR34a* under salt stress conditions. We treated wild-type grape callus with 100 mM NaCl for various periods of time, and then extracted and reverse transcribed RNA from the callus for qRT-PCR analysis. *VvActin* was used as the reference gene for qRT-PCR normalization. The analysis results showed that the relative level of transcripts of *VvSR34a* showed a trend of increasing at first and then decreasing under salt stress treatment, and reached the maximum value at 2 h of salt treatment (Figure 1). These results suggest that *VvSR34a* may be involved in the regulatory response of grape to salt stress.

### 2.2. Subcellular Localization of VvSR34a Gene

**Figure 2:**
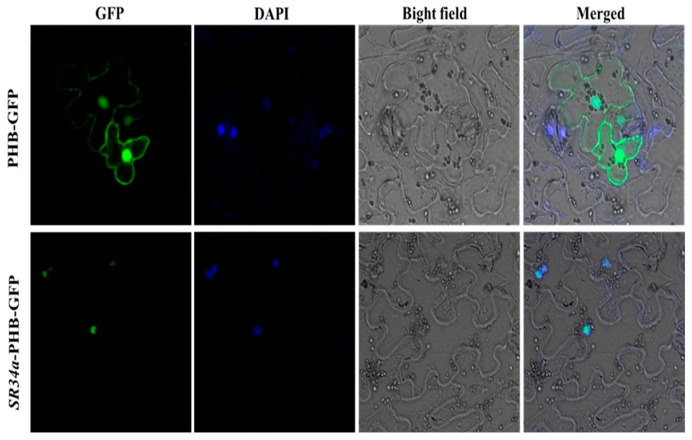
Plant subcellular localization analysis of *VvSR34a*. This nuclear localization result was verified through three independent replicate experiments, and consistent localization patterns were observed in each trial. Green represents GFP fluorescence, while blue represents DAPI-stained cell nuclei.

We inserted the *VvSR34a* coding sequence into the PHB-GFP vector to generate a fusion construct with GFP. The recombinant plasmid *SR34a*-PHB-GFP and empty PHB-GFP vector were introduced into tobacco (*Nicotiana benthamiana*) leaves for co-expression by Agrobacterium-mediated transient transformation of tobacco. After 2 days of culture, the leaves were harvested and observed under laser confocal microscope. The fluorescence signal showed that *VvSR34a* was located in the nucleus (Figure 2).

### 2.3. Overexpression of VvSR34a Enhances Salt Tolerance of Grapes

**Figure 3:**
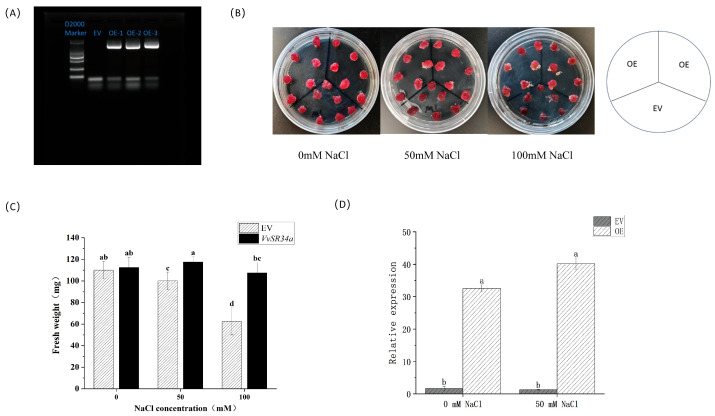
Phenotype of *VvSR34a*-transgenic callus under salt stress: (**A**) PCR gel map of positive callus. Amplification was performed using the R primer of the PHB-GFP vector and the F primer of the *VvSR34a* CDS sequence. (**B**) Phenotypic analysis of overexpression and empty at different salt concentration. EV represents callus infected with empty vector, and OE represents overexpression callus. (**C**) Comparison of fresh weight between overexpression callus and empty vector callus under salt stress. (**D**) Analysis of *VvSR34a* expression under salt stress. Each value represents the average ± SD of three independent biological replicates. The significant difference between means (*p* < 0.05) is represented by different letters.

The expression of *VvSR34a* was induced by salt stress, suggesting that *VvSR34a* may regulate the salt stress response of plants. In order to further explore the function of *VvSR34a* in grapes, we introduced the recombinant plasmid *SR34a*-PHB-GFP into grape callus by Agrobacterium-mediated transformation. Hygromycin was added to B5 medium to select positive callus, and their identity was confirmed by extraction of callus DNA and PCR verification (Figure 3A). We then observed the phenotype of the overexpressed callus cultured in B5 medium containing 0 mM, 50 mM and 100 mM NaCl for 14 days, and the callus overexpressing *VvSR34a* grew better under salt stress than those without NaCl (Figure 3B). In addition, under salt stress, the fresh weight of the empty vector callus was significantly reduced, showing severe stress damage, whereas the fresh weight of the overexpression callus changed slightly, indicating higher salt tolerance (Figure 3C). qRT-PCR analysis showed that *VvSR34a* was up-regulated under salt stress (Figure 3D).

**Figure 4:**
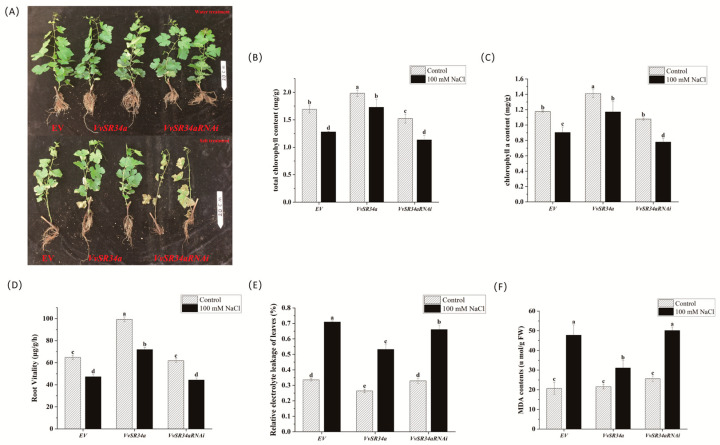
Phenotypic analysis of *VvSR34a*-transgenic cuttings under salt stress. (**A**) Phenotypic images of transgenic cuttings under salt treatment, scale bar = 20 cm. (**B**) Total chlorophyll content of cuttings under salt treatment. (**C**) Chlorophyll a content of cuttings under salt treatment. (**D**) Root activity of overexpression and EV grape plants under salt stress. (**E**) Leaf electrolyte leakage of overexpression and EV grape plants under salt stress. (**F**) Malondialdehyde (MDA) content in leaves of overexpression and EV grape plants under salt stress. Each value represents the average ± SD of three independent biological replicates. The significant difference between means (*p* < 0.05) is represented by different letters.

To investigate the salt tolerance functions of *VvSR34a* in grape seedlings, we further constructed *VvSR34aRNAi* silencing vectors. Annual cuttings were used for the cutting experiment, and four-week-old rooted cuttings were subjected to 100 mM NaCl treatment. Phenotypic observations were conducted after three weeks of treatment. The results showed that the growth performance of *VvSR34a*-overexpressing lines was significantly better than that of the empty vector control, whereas the silencing of *VvSR34a* resulted in higher sensitivity to salt stress compared with the control (Figure 4A). Under normal water treatment, no significant differences in total chlorophyll and chlorophyll a contents were observed among all lines. However, after salt treatment, the contents of total chlorophyll and chlorophyll a in all lines were significantly decreased. Notably, the *VvSR34a* overexpression (OE) lines showed the smallest reduction in chlorophyll content, which remained significantly higher than that of the empty vector (EV) control lines, while the *VvSR34a*-RNAi lines exhibited the largest reduction in chlorophyll content, which was significantly lower than that of the empty vector (EV) control lines (Figure 4B,C). Root activity is a crucial indicator reflecting root absorption capacity and stress resistance in plants. Under salt stress, plant roots activate intrinsic defense mechanisms to cope with adverse conditions. After salt stress treatment, the root activity of grape seedlings exhibited a declining trend. Importantly, the root activity of *VvSR34a* OE lines was significantly higher than that of both EV and *VvSR34a*-RNAi lines, while no significant difference was detected between the EV and *VvSR34a*-RNAi groups. These findings indicate that *VvSR34a* positively regulates root activity under salt stress (Figure 4D). Leaf relative electrolyte leakage (REL) and malondialdehyde (MDA) content are key indicators reflecting the degree of plant cell membrane damage. Salt stress disrupts the integrity of cell membranes, leading to increased electrolyte leakage, the accumulation of reactive oxygen species (ROS), and elevated MDA synthesis (a marker of lipid peroxidation). After salt stress treatment, leaf REL was significantly increased in all lines, but the magnitude of increase varied significantly among groups. The EV control and *VvSR34a*-RNAi lines showed a much greater increase in REL, accompanied by a significant elevation in MDA content, indicating severe cell membrane damage and high levels of lipid peroxidation in these lines (Figure 4E,F). These results showed that the overexpression of *VvSR34a* could enhance the salt tolerance of grapes.

### 2.4. Physical Interaction of VvSR34a with VvCOP9

**Figure 5:**
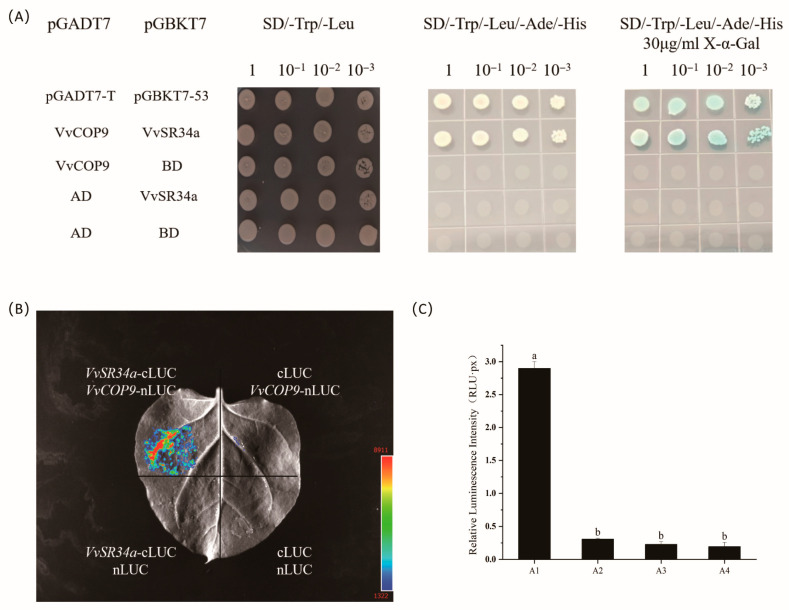
Physical interaction of VvSR34a with VvCOP9: (**A**) Y2H indicates that VvCOP9 interacts with VvSR34a. Among them, pGADT7-T and pGBKT7-53 served as the positive control. (**B**) The LUI experiment show that VvSR34a and VvCOP9 interact in plant cells. cLUC + nLUC served as the negative control. (**C**) Relative Luminescence Intensity. A1: *VvSR34a*-Cluc + *VvCOP9*-nLUC, A2: cLUC + *VvCOP9*-Nluc, A3: *VvSR34a*-Cluc + nLUC, A4: cLUC + nLUC. Each value represents the average ± SD of three independent biological replicates. The significant differences between means (*p* < 0.05) are represented by different letters.

In order to elucidate the regulatory mechanism of *VvSR34a* in response to salt stress, we screened the proteins interacting with *VvSR34a* by yeast two-hybrid (Y2H). *VvSR34a* was used as bait protein to screen the cDNA library of *Vitis vinifera*. The COP9 (Constitutive Photomorphogenetic 9) Signalome (CSN) is an eight-subunit protein complex that is conserved throughout evolution and mediates light-regulated development and stress-linked resistance in plants [18]. To further clarify the interaction between these two proteins, we co-transformed yeast strains *VvSR34a*-pGBKT7 and *VvCOP9*-pGADT7 on SD/-Trp/-Leu and SD/-Trp/-Leu/-Ade/-His-deficient media and found that they grew normally, indicating that the two interacted within the yeast cells (Figure 5A). In order to further verify the interaction in plants, *VvSR34a* was fused to the C-terminus (cLUC) of luciferase and *VvCOP9* was fused to the N-terminus (nLUC) of luciferase and co-transformed into tobacco leaves using luciferase complementation (LCI) assay. Strong fluorescence signals from the injection site of tobacco leaves were observed under the plant in vivo imaging system (Figure 5B). We further performed a quantitative analysis of the luminescence intensity in the LCI assay. The results showed that the luminescence intensity of the A1 group was significantly higher than other groups (Figure 5C). Taken together, these results further confirmed the direct interaction between VvSR34a and VvCOP9.

### 2.5. Phenotypic Analysis of VvCOP9-Transgenic Cutting Seedlings Treated with Salt

**Figure 6:**
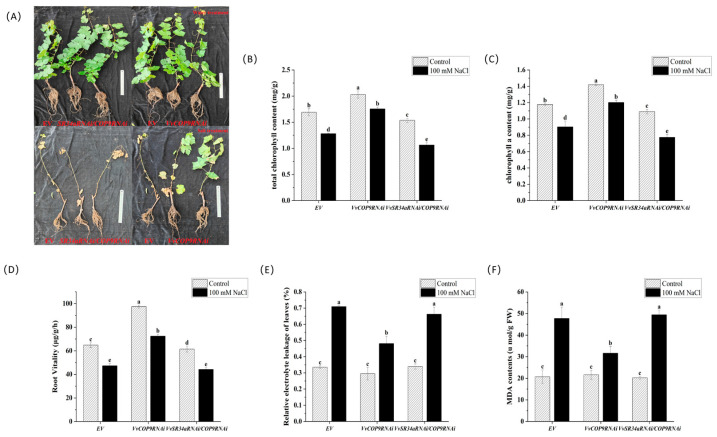
Phenotypic analysis of *VvCOP9*-transgenic cuttings under salt stress. (**A**) Phenotypic images of transgenic cuttings under salt treatment, scale bar = 20 cm. (**B**) Total chlorophyll content of cuttings under salt treatment. (**C**) Chlorophyll a content of cuttings under salt treatment. (**D**) Root activity of overexpression and EV grape plants under salt stress. (**E**) Leaf electrolyte leakage of overexpression and EV grape plants under salt stress. (**F**) Malondialdehyde (MDA) content in leaves of overexpression and EV grape plants under salt stress. Each value represents the average ± SD of three independent biological replicates. The significant difference between means (*p* < 0.05) is represented by different letters.

Additionally, we also constructed the *VvCOP9RNAi* silencing vector and *VvSR34aRNAi/VvCOP9RNAi* double silencing vector. Annual cuttings were used for the cutting experiment. The results showed that the growth performance of *VvCOP9*-RNAi lines was significantly better than that of the empty vector control, whereas the silencing of *VvSR34a/VvCOP9* resulted in higher sensitivity to salt stress compared with the control (Figure 6A). After salt stress treatment, the contents of total chlorophyll and chlorophyll a in *VvCOP9*-RNAi lines were significantly higher than those in the empty vector (EV) control (Figure 6B,C). Subsequently, root vitality assays were performed on transgenic lines. The root activity of *VvCOP9*-RNAi lines was significantly higher than that in the other two lines, suggesting their stronger tolerance to stress (Figure 6D). After exposure to salt stress, leaf electrolyte leakage significantly increased in all lines compared with the water control. Specifically, electrolyte leakage was significantly elevated in the empty vector (EV) and *VvSR34a/VvCOP9*-RNAi lines, whereas only a slight increase was observed in the *VvCOP9*-RNAi lines (Figure 6E). In addition, leaf malondialdehyde (MDA) content was notably enhanced following salt stress treatment. The *VvCOP9*-RNAi lines showed a much smaller increment in MDA content, which suggested that this line possessed higher salt tolerance (Figure 6F). Under salt stress, the *VvCOP9*-RNAi lines exhibited vigorous growth with milder leaf wilting symptoms, showing a salt-tolerant phenotype similar to that of the *VvSR34a*-overexpressing lines. In contrast, the *VvSR34a/VvCOP9*-RNAi lines suffered severe damage with leaf necrosis and abscission, a phenotype analogous to that of the *VvSR34a*-RNAi lines. These observations indicated that silencing *VvCOP9* significantly enhanced the salt tolerance of grapevine, suggesting that *VvCOP9* may play a negative regulatory role in the salt stress response of grapevine. Meanwhile, the loss of the high salt-tolerant phenotype in the double-silenced lines demonstrated that *VvCOP9* is genetically upstream of *VvSR34a*.

### 2.6. VvCOP9 Mediates the Ubiquitination-Dependent Proteasomal Degradation of VvSR34a

**Figure 7:**
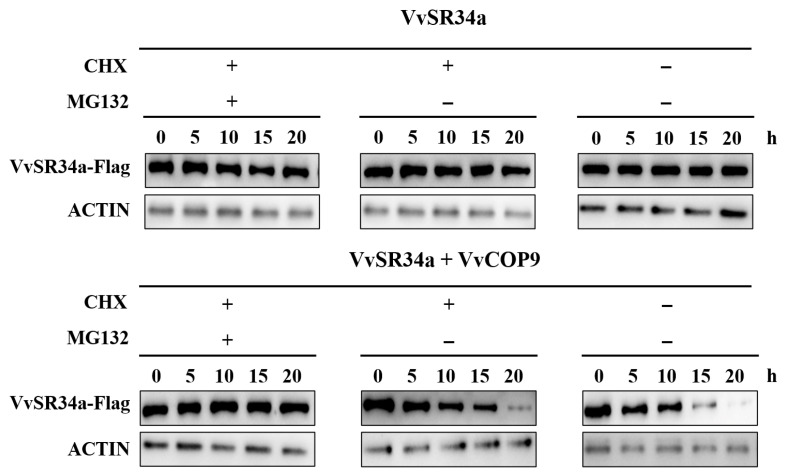
Overexpression of VvCOP9 promotes the ubiquitin-mediated degradation of VvSR34a protein. CHX: cycloheximide; it blocks protein synthesis, ensuring that the detection system exclusively reflects the degradation of pre-existing proteins. MG132: a specific inhibitor of the 26S proteasome, which completely blocks protein degradation mediated by the ubiquitin–proteasome pathway.

In grapevines, *VvCOP9* is genetically positioned upstream of *VvSR34a*. *VvSR34a* positively regulates the salt stress response in grapevines, while *VvCOP9* exerts a negative regulatory function in this process. Based on the above genetic hierarchical relationship and functional correlation, we hypothesized that VvCOP9 may promote the degradation of VvSR34a via ubiquitination modification. To verify this hypothesis, we constructed recombinant expression vectors carrying VvSR34a-Flag and VvCOP9-HA, and performed Agrobacterium-mediated co-infiltration of *Nicotiana benthamiana* leaves. Samples were collected at sequential time points for total protein extraction, and the abundance of VvSR34a-Flag was detected by Western blot assay. When VvSR34a was overexpressed alone, the degradation rate of VvSR34a was extremely slow after cycloheximide (CHX) treatment, indicating that the protein itself has high stability in plant cells. When VvSR34a was co-expressed with VvCOP9, VvSR34a protein was significantly degraded with prolonged treatment time in the CHX-only treatment group. In contrast, this degradation effect was completely abolished in the group co-treated with CHX and MG132, and the protein abundance remained stable (Figure 7). The above results demonstrated that VvCOP9 can significantly promote the ubiquitination-dependent degradation of VvSR34a, and this degradation effect is dependent on the 26S proteasome pathway. Inhibition of the proteasome by MG132 can directly abolish this degradation process.

### 2.7. Involvement of VvSR34a in the Expression of Salt-Responsive Genes

**Figure 8:**
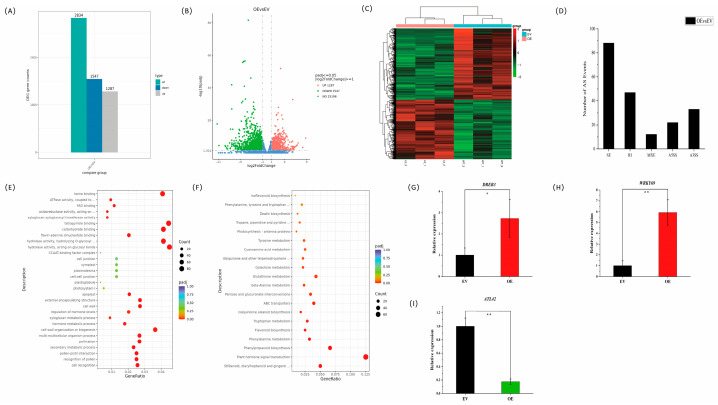
*VvSR34a* differential relative level of transcripts analysis: (**A**) bar chart of the number of differentially expressed genes between OE and EV. Blue and gray represent up-regulated and down-regulated genes, respectively. Numbers on the bars indicate the number of differentially expressed genes. (**B**) Volcano plot of differentially expressed genes between OE and EV. The *x*-axis shows log2FoldChange values, and the *y*-axis represents −log10padj or −log10pvalue. Blue dashed lines indicate the threshold for screening differentially expressed genes. (**C**) Cluster heatmap of differentially expressed genes between OE and EV. The *x*-axis represents sample names, and the *y*-axis shows normalized FPKM values of differentially expressed genes. Redder color indicates higher expression level, and greener color indicates lower expression level. (**D**) Statistics of alternative splicing (AS) events between OE and EV. (**E**) Scatter plot of GO enrichment analysis. The *x*-axis represents the ratio of the number of differentially expressed genes annotated to each GO term to the total number of differentially expressed genes, and the *y*-axis represents the GO term. (**F**) Scatter plot of KEGG enrichment analysis. The *x*-axis represents the ratio of the number of differentially expressed genes annotated to each KEGG pathway to the total number of differentially expressed genes, and the *y*-axis represents the KEGG pathway. (**G**–**I**) qRT-PCR analysis of the expression levels of marker genes involved salt response pathways. Data are represented as means ± SD of three replicates. (* *p* < 0.05, ** *p* < 0.01).

To understand the transcriptional changes in *VvSR34a*-overexpressing callus during salt stress, we performed transcriptome analysis using transgenic callus (OE) and empty controls (EV) treated with salt for 9 days and sequenced in three biological replicates. Analysis of the transcriptome data revealed 2834 differentially expressed genes in OE compared to EV (standard: |log2(FoldChange)| ≥ 1 and padj ≤ 0.05), of which 1287 genes were up-regulated and 1547 gene were down-regulated (Figure 8A,B). Cluster analysis of differentially expressed genes revealed that many genes down-regulated in EV showed higher expression in OE (Figure 8C). Further analysis of the RNA-seq data revealed that, compared with EV, 202 alternative splicing events were detected in OE, including 88 skipped exons (SE), 47 retained introns (RI), 12 mutually exclusive exons (MXE), 22 alternative 5′ splice sites (A5SS), and 33 alternative 3′ splice sites (A3SS) (Figure 8D). Gene ontology and the Kyoto Encyclopedia of Genes and Genomes analysis indicate that these genes with altered expression in OE are involved in different biological process, including ATPase activity, oxidoreductase activity, and phytohormone signaling pathways (Figure 8E,F). To validate the transcriptome (RNA-seq) analysis, the expression levels of differentially expressed genes were determined by qRT-PCR. Consistent with the RNA-seq data, positive regulators implicated in the salt stress response pathway, including dehydration-responsive element-binding protein 3 (*DREB3*) and WRKY transcription factor 69 (*WRKY69*), were significantly up-regulated in *VvSR34a*-overexpressing lines (Figure 8G,H). In contrast, the negative salt stress regulator E3 ubiquitin-protein ligase *ATL42* was markedly down-regulated in the *VvSR34a*-overexpressing lines (Figure 8I). Collectively, these results demonstrate that *VvSR34a* functions in response to salt stress in grapes.

## 3. Discussion

Salt stress is one of the main abiotic stresses in global agriculture, which seriously affects the growth and development of plants [19]. In order to adapt and tolerate salt stress that may threaten their survival throughout their life cycle, many plants have evolved a variety of strategies [20]. It has been reported that a large number of SR proteins in different species are induced and functionally differentiated when subjected to abiotic stress [21]. In this study, *VvSR34a* was deeply explored, and it was clear that it played a key role in the regulation of salt tolerance in grapes. This study found that the relative level of *VvSR34a* showed a ‘rise first and then decline’ pattern under 100 mM NaCl treatment, reaching the peak expression level at 2 h after treatment (Figure 1). This dynamic expression pattern is consistent with that of typical early stress-responsive genes in plants, suggesting that *VvSR34a* may act as an early response factor to salt stress and participate in the initial transduction of stress signals. For example, in *Vitis vinifera*, the relative level of the transcription factor *VvCBF4* is significantly induced by salt and low-temperature stress and enhances salt tolerance by regulating hormonal signaling and antioxidant pathways [22]. In addition, it has been confirmed that several RNA-binding protein (RBP) genes in grapes exhibit similar induced expression patterns under salt stress, and nuclear localized RBP can regulate stress response pathways by regulating the splicing efficiency of transcription factor mRNA. For example, the expression of the *VvRBP18* gene in grapes reaches its peak at 3 h of salt stress, and is also located in the nucleus, which regulates its splicing efficiency by specifically binding to the mRNA precursor of WRKY transcription factor, thus affecting the expression pattern of salt-tolerance-related genes [23].

Grape callus overexpressing *VvSR34a* exhibited significantly better growth status under 100 mM NaCl stress than the empty vector control, with a smaller reduction in fresh weight (Figure 3). In the cutting seedling experiment, plants overexpressing *VvSR34a* showed higher chlorophyll content, stronger root activity, and lower electrolyte leakage and MDA content under salt stress (Figure 4), which directly confirmed that *VvSR34a* positively regulates salt tolerance in grapes. In contrast, the silencing of *VvSR34a* significantly increased the sensitivity of grapes to salt stress, further verifying the salt tolerance regulatory function of this gene. These results are consistent with the functional studies of SR proteins such as *AtSR45a* in Arabidopsis and *OsSCL30* in rice under abiotic stress, indicating that SR proteins have conserved functions in plant salt tolerance regulation [11,13]. Yeast two-hybrid and luciferase complementation assays confirmed that VvSR34a physically interacts with VvCOP9 (Figure 5). *VvCOP9* serves as a core subunit of the COP9 signalosome (CSN), an evolutionarily highly conserved multifunctional regulatory complex that is ubiquitous across eukaryotes. In plants, this complex has been well documented to play fundamental roles in regulating diverse biological processes, including growth, development and abiotic stress responses. Furthermore, silencing of *VvCOP9* also enhanced salt tolerance in grapes, suggesting that *VvCOP9* acts as a negative regulator of salt stress (Figure 6). Meanwhile, the loss of the high salt-tolerant phenotype in the double-silenced lines demonstrated that *VvCOP9* is genetically upstream of *VvSR34a*. To test the regulatory relationship between these two proteins, we conducted an in vivo ubiquitination and degradation assay. Our results revealed that the co-expression of VvSR34a and VvCOP9 markedly accelerated the degradation rate of VvSR34a protein. Furthermore, this degradative effect was completely abolished by co-treatment with the 26S proteasome inhibitor MG132 (Figure 7). The COP9 signalosome is an evolutionarily highly conserved eight-subunit complex whose core function is involved in regulating protein degradation via the ubiquitin–proteasome pathway. Previous studies have shown that the COP9 signalosome participates in plant stress responses by mediating the ubiquitination and degradation of target proteins [24,25]. Based on the results of this study, we speculate that *VvCOP9* may bind to *VvSR34a* and promote its ubiquitination and degradation, thereby reducing the protein abundance of *VvSR34a* and inhibiting the activation of salt-tolerant pathways.

Transcriptome analysis revealed that there were 2834 differentially expressed genes and 202 alternative splicing events in callus overexpressing *VvSR34a*. These differentially expressed genes were significantly enriched in ATPase activity, oxidoreductase activity, and plant hormone signal transduction pathways (Figure 8). Among them, positive regulators of salt tolerance such as dehydration-responsive element-binding protein 3 (*DREB3*) [26] and WRKY transcription factor 69 (*WRKY69*) [27] were significantly up-regulated in the overexpression lines, while negative regulators of salt stress such as E3 ubiquitin-protein ligase ATL42 were significantly down-regulated [28]. These results were consistent with the qRT-PCR verification. DREB transcription factors are classic salt tolerance regulators in plants, which can activate the expression of downstream salt-tolerant genes by binding to DRE elements. WRKY transcription factors participate in salt tolerance regulation by modulating oxidative stress responses and hormone signaling pathways [29,30]. In addition, the differentially expressed genes were also enriched in pathways related to ATPase activity. ATPases can alleviate ion toxicity under salt stress by maintaining ion homeostasis inside and outside cells, suggesting that *VvSR34a* may also participate in the maintenance of ion homeostasis under salt stress by regulating the expression of ion transport-related genes. The present study only characterized the changes in differentially expressed genes under salt stress. As a splicing factor, it remains largely unknown which specific downstream target genes are spliced by *VvSR34a* to exert its biological function. Nevertheless, our findings lay a solid foundation for further improving the detailed regulatory mechanism of this gene in subsequent studies.

## 4. Materials and Methods

### 4.1. Plant Material and Growth Conditions

In this experiment, the experimental materials used included grape cuttings, ‘red Gamay’ grape callus and tobacco. The grape cuttings were obtained from annual high-quality branches of Cabernet Sauvignon. They were propagated cuttings in spring, and grown in nutrient soil with a 1:1 (*v*/*v*) mixture of sod soil and vermiculite. When the seedlings developed 6–8 fully expanded mature functional leaves, they were subjected to subsequent experiments. Red Gamay grape callus were cultured aseptically at 24 °C for 16 h/8 h (light/dark) and subcultured every 3 weeks. The basal medium comprised B5 salts at 3.16 g/L, sucrose at 20 g/L, myo-inositol at 0.2 g/L, and agar at 7 g/L. Tobacco (*Nicotiana benthamiana*) was grown in a growth chamber (light/dark, 16 h/8 h) at 25 °C.

### 4.2. Gene Cloning and Vector Construction

Total RNA was isolate from grape callus using that RNAprep pure Plant kit (Tanon biotechnology, Shanghai, China, DP441). After DNase I digestion, complementary DNA (cDNA) was synthesized by reverse transcription using a PrimeScript RT kit (TaKaRa, Dalian, China, RR047A) containing a gDNA eraser. The resulting cDNA was used as a template, and the coding DNA sequence of the *VvSR34a* gene was acquired by PCR amplification using PrimeSTAR^®^ Max DNA Polymerase (TaKaRa). The amplified products were purified by using the FastPure Gel DNA Extraction Mini Kit (Vazyme Biotech Co., Ltd., Nanjing, China). The recovered gene fragments were ligated into the PHB-GFP plant expression vector using ClonExpress MultiS One Step Cloning Kit (Vazyme). The recombinant vector was then transformed into E-coli DH5α competent cells. Colony PCR was performed using 2× Rapid Taq Master Mix (Vazyme Biotech Co., Ltd., Nanjing, China) for validation. Plasmid extraction was conducted using the FastPure Plasmid Mini Kit (Vazyme Biotech Co., Ltd., Nanjing, China). *Agrobacterium* competent cells were purchased from Shanghai Weidi Biotech (Shanghai, China) Co., Ltd. The previously constructed expression vector was transformed into *Agrobacterium* GV3101 and LB4404 via the freeze–thaw method for subsequent genetic transformation. The primers used for vector construction are provided in the Appendix A (Table A1).

### 4.3. Subcellular Localization

To determine the subcellular localization of *VvSR34a*, the *VvSR34a* coding sequence was cloned into the PHB-GFP vector to generate the VvSR34a-PHB-GFP fusion construct. The fusion vector and empty PHB-GFP (control) were transformed into *Agrobacterium* strain GV3101 and transiently expressed in 4-week-old *Nicotiana benthamiana* leaves via Agrobacterium-mediated infiltration (adjust the optical density (OD600) of the *Agrobacterium* suspension to 0.6–0.8). After 24–48 h incubation (25 °C, 16 h light/8 h dark), leaf lower epidermis was peeled, counterstained with DAPI (5 min) to label nuclei, and observed under a laser confocal microscope. GFP and DAPI signals were detected. Experiments were performed with three independent biological replicates, and at least 10 cells were analyzed per replicate.

### 4.4. Analysis of Relative Level of Transcripts

Total plant RNA was extracted using the RNAprep Pure Polysaccharide and Polyphenol Plant Total RNA Extraction Kit (DP441, Tiangen Biotech, Beijing, China). First-strand cDNA was synthesized from 1 μg of total RNA using the PrimeScript™ RT reagent Kit with gDNA Eraser (Perfect Real Time, TaKaRa, Dalian, China), following the manufacturer’s instructions to remove genomic DNA contamination. Quantitative real-time polymerase chain reaction (qRT-PCR) was performed using ChamQ™ Universal SYBR qPCR Master Mix (Vazyme, Nanjing, China, Cat. No. 7E280C8) on a real-time PCR detection system. The reaction conditions were set according to the manufacturer’s protocol. For each sample, three independent biological replicates were analyzed, with each biological replicate subjected to three technical replicates to ensure the reliability of the results. The grape *VvActin* gene was used as the internal reference for normalization of relative level of transcripts.

### 4.5. Transformation of Grape Callus

A single colony of *Agrobacterium tumefaciens* strain LB4404 harboring the expression vector was picked from the frozen stock, streaked on agar plates for recovery, and inoculated into 20 mL of liquid LB medium, followed by activation at 28 °C on a shaker for 20–24 h. Then, 1 mL of the primary activated culture was transferred into 50 mL of liquid LB medium supplemented with antibiotics (Kanamycin, Rifampicin) and cultured at 28 °C for 10–12 h until the culture turned golden yellow. The bacterial cells were collected by transferring the activated culture into a 50 mL centrifuge tube and centrifuging at room temperature for 10 min. Under sterile conditions, the pellet was resuspended in sterile water and centrifuged again at 7155× *g* for 5 min to remove residual LB medium; this washing step was repeated. The cells were finally resuspended in solution (4.44 g/L MS salts, 30 g/L sucrose), and the OD_600_ was adjusted to 0.6–0.8, followed by activation at 28 °C for 30 min. Calli cultured for approximately 20 days with good vigor were selected and immersed in the bacterial suspension for 20 min. After rinsing with sterile water, the callus were placed on co-cultivation medium and incubated in darkness at 25 °C for 2 days. The callus were then transferred to delay medium (3.16 g/L B5 salts, 20 g/L sucrose, 0.2 g/L inositol, 300 mg/L cefotaxime, and 7 g/L agar) and cultured in darkness at 25 °C for 20–30 days. Once new callus emerged on the delay medium, they were transferred to selection medium (3.16 g/L B5 salts, 20 g/L sucrose, 0.2 g/L inositol, 300 mg/L cefotaxime, 50 mg/L hygromycin, and 7 g/L agar), with subculture every 20 days. Genomic DNA was extracted from callus after three rounds of selection, and PCR amplification was performed using the gene-specific forward primer and the vector reverse primer to verify the transgenic callus.

### 4.6. Salt Stress

Salt-based plate treatment: Wild-type and transgenic callus were transferred to the MS medium supplemented with 50 mM and 100 mM NaCl, respectively, and cultured for 14 days, while the control group was normal growth medium, and the growth changes were observed; each treatment was biologically repeated in triplicate.

Salt treatment of cutting seedlings: When the grape cutting seedlings grown in pots developed 8–10 new intact leaves, seedlings with consistent growth performance were selected for salt stress treatment. Salt stress was imposed by irrigating each seedling with 500 mL of 100 mM NaCl solution, while 500 mL of distilled water was used as the control. Both treatments were conducted every 3 days for a total duration of 21 days, with three biological replicates set for each treatment to ensure the reliability of the experimental results.

Determination of total chlorophyll content [31]: Weigh 0.2 g of fresh plant leaves (avoiding leaf veins), rinse it thoroughly, dry it, and cut it into small pieces. Transfer the minced tissue into a 50 mL centrifuge tube, add 10 mL of 95% ethanol, and perform dark extraction for 24 h. After 24 h, dilute the extract to the 25 mL mark with 95% ethanol, and measure the absorbance at the wavelengths of 470 nm, 649 nm, and 665 nm. The formula for determining chlorophyll content is as follows:

Ca = 13.95 × (OD665)6.88 × (OD649)

Cb = 24.96 × (OD649)7.32 × (OD665)

C = Ca + Cb

Chloroplast pigment content (mg/g) = C × V × r/(1000 × m),

Ca: concentration of chlorophyll a (mg/L), Cb: concentration of chlorophyll b (mg/L), C: concentration of total chlorophylls (mg/L), V: volume of the extract (mL), r: dilution factor, m: sample mass (g).

Determination of root vitality: Root vitality was determined using the triphenyltetrazolium chloride (TTC) method [31]. (1) Exactly 0.5 g of fresh fine roots was weighed, cut into approximately 1 cm segments, and placed into a 50 mL centrifuge tube. A total of 10 mL of a pre-mixed 1:1 solution of TTC staining solution and phosphate buffer was added to the sample. For the control group, an equal weight of root tissue was treated with 10 mL of a mixture composed of 1 mol/L sulfuric acid and the same 1:1 TTC-phosphate buffer solution. (2) The tubes were incubated in a water bath at 38 °C in the dark for 4 h. (3) After incubation, 3 mL of 1 mol/L sulfuric acid was added to terminate the reaction. (4) The tubes were gently inverted and mixed to accelerate the termination. After 20 min, the reaction solution was removed, and 20 mL of 95% ethanol was added. The tubes were then incubated at room temperature in the dark for 24 h to extract. (5) The absorbance of the extract was measured at 485 nm, with the control sample used for background correction.

Determination of leaf electrolyte leakage in plants [31]: Leaf electrolyte leakage was measured using an FE30 conductivity meter. Samples were washed with deionized water and dried, then sliced into 5 mm diameter disks. After thorough mixing, 0.5 g of disks was transferred into a 25 mL test tube, and 15 mL of deionized water was added. The test tube was placed on a shaker at 25 °C and shaken for 2 h until the leaf disks sank to the bottom, and the initial electrical conductivity (R1) was recorded. Subsequently, the sample was boiled in boiling water for 30 min, then allowed to cool to room temperature, and the final conductivity R2 was measured.

Electrolyte leakage rate (%) = [(R1 − Blank)/(R2 − Blank)] × 100%

Malondialdehyde (MDA) content was determined using the thiobarbituric acid (TBA) chromogenic method. (1) Fresh plant tissue (0.5 g) was ground in a mortar pre-chilled at 4 °C. During homogenization, 5 mL of pre-prepared 10% trichloroacetic acid (TCA) was added to the mortar in two batches. (2) After complete homogenization, the homogenate was transferred into a 10 mL tube and centrifuged at 2795× *g* for 10 min. (3) A 2 mL aliquot of the supernatant was transferred to a new 10 mL tube, and 2 mL of TBA solution was added. (4) The mixture was incubated in a boiling water bath for 20 min, then quickly cooled in ice water. After cooling to room temperature, the mixture was centrifuged at 2795× *g* for 10 min. (5) The supernatant was collected, and its absorbance was measured at 450 nm, 532 nm, and 600 nm.

MDA concentration (μmol/g FW) = [6.45 × (A532 − A600) − 0.56 × A450] × V/W

A450, A532, A600: Absorbance at 450 nm, 532 nm, and 600 nm, respectively

V: Total volume of the extract (mL)

W: Fresh weight of the sample (g)

### 4.7. Protein–Protein Interactions

Yeast two-hybrid experiment (Y2H): In this experiment, the *VvSR34a* gene and *VvCOP9* gene were cloned into pGADT7 and pGBKT7 vector, respectively, by the co-expression of pGADT7 and pGBKT7 in Y2H gold. The two vectors were co-transformed into yeast strains, and positive clones were screened on SD/-Trp/-Leu and SD/-Trp/-Leu/-Ade/-His deficient medium (YNB, ammonium sulfate, glucose, amino acids). Luciferase complementation assay (LCI) [32]: *VvSR34a* was fused to the C-terminal (cLUC) of luciferase, and *VvCOP9* was fused to the N-terminal (nLUC) of luciferase to construct the corresponding plant expression vectors. The method comprises the following steps: infiltrating and injecting Agrobacterium containing the fusion expression vector into *Nicotiana benthamiana* leaves, uniformly coating a luciferase substrate on an injection part after 2–3 days, and detecting the bioluminescence intensity by using a plant living body imaging system to judge whether interaction exists between proteins.

### 4.8. Ubiquitination Degradation Assay

The VvSR34a-Flag and VvCOP9-HA vectors were constructed and transformed into Agrobacterium. The plasmids were then co-transformed into tobacco leaves via Agrobacterium-mediated transformation, with the co-transformation of VvSR34a-Flag and the empty HA vector serving as the control group. After infiltration, the plants were cultured normally for one day to allow sufficient expression of the exogenous proteins. Transformed tobacco leaves were ground in liquid nitrogen. A 0.5 g aliquot of the powder was transferred into a 5 mL centrifuge tube, and 2–3 mL of protein extraction buffer was added. After thorough vortexing, the mixture was incubated at 4 °C for 1 h to lyse the proteins (Nuclear and Cytoplasmic Protein Extraction Kit, Beyotime, Shanghai, China). The protein extract was transferred into two 1.5 mL centrifuge tubes and centrifuged at 13,680× *g* for 10 min at 4 °C. The supernatant was transferred to a new 1.5 mL centrifuge tube and centrifuged again under the same conditions. Then, 50 μL of 1× SDS-PAGE loading buffer was added, and the samples were heated at 95 °C for 10 min. After centrifugation, the samples were subjected to Western blot analysis. The separating gel and stacking gel were prepared according to the instructions of the SDS-PAGE gel kit (CW0022M, CWBIO, Beijing, China). Electrophoresis was performed at 120 V until the marker bands reached the bottom of the separating gel. The PVDF membrane was activated by soaking in methanol for 2–3 min. The transfer sandwich was assembled from the cathode to the anode in the order of sponge, filter paper, gel, PVDF membrane, filter paper, and sponge. The assembled cassette was placed into the transfer tank with transfer buffer, and electroblotting was conducted at a constant current of 0.4 A for 40 min. The PVDF membrane was incubated in blocking solution on a shaker for 10 min. Primary antibody was diluted at an appropriate ratio in 1× TBST containing 5% (*w*/*v*) non-fat dry milk. After discarding the blocking solution, the membrane was incubated with primary antibody overnight. The membrane was then washed with 1× TBST four times for 6 min each. Subsequently, the membrane was incubated with secondary antibody on a shaker for 1 h, followed by four washes with 1× TBST for 6 min each. Finally, the PVDF membrane was placed in a square dish, incubated with ECL detection reagent, and visualized and photographed using a chemiluminescence imaging system.

### 4.9. RNA-Seq Analysis

Transgenic (OE) and empty (EV) grape callus were treated with 50 mM NaCl for one week in three independent replicates per sample. The transcriptome sequencing experiment was completed by Novogene Co., Ltd. (Beijing, China). The technical workflow of RNA-seq primarily consists of two parts: library preparation and sequencing, and bioinformatics analysis. First, RNA is extracted from tissues or cells, followed by stringent quality control of the RNA samples, mainly performed using the Agilent 2100 bioanalyzer (Agilent Technologies, Inc. Santa Clara, CA, USA) to accurately assess RNA integrity. Oligo dT magnetic beads are used to enrich mRNA from total RNA. After mRNA fragmentation, first-strand cDNA is synthesized using random hexamer primers, followed by second-strand cDNA synthesis. After end repair, A-tailing, adapter ligation, fragment selection, amplification, and purification, the sequencing library is constructed. Once the library passes quality control, different libraries are pooled according to their effective concentration and the required data output, and then subjected to Illumina sequencing. In the sequencing flow cell, four types of fluorescently labeled dNTPs, DNA polymerase, and adapter primers are added for amplification. During the extension of the complementary strand in each sequencing cluster, each added fluorescently labeled dNTP releases a corresponding fluorescence signal. The sequencer captures the fluorescence signals and, using computer software, converts the optical signals into sequencing peaks, thereby obtaining the sequence information of the target fragments. The reference genome ID for the grape library is ensembl_53_vitis_vinifera_pn40024_v4_toplevel. The core of RNA-seq is the significance analysis of gene expression differences, which uses statistical methods to compare gene expression differences between two or more conditions, identify specific genes associated with the conditions, and then further analyze the biological significance of these specific genes. The screening criteria for differentially expressed genes is |log2(FoldChange)| ≥ 1 and padj ≤ 0.05. The analysis process includes quality control, alignment, quantification, significance analysis of differential expression, and functional enrichment. In addition, alternative splicing, variant site detection, and novel transcript prediction are also important analytical components of RNA-seq.

## 5. Conclusions

In this study, we found that *VvSR34a* is a positive regulator of salt stress response in grapevines. It showed an expression pattern of ’first increase then decrease’ under 100 mM NaCl treatment, with the expression peak at 2 h, and was localized in the nucleus. The overexpression of *VvSR34a* significantly enhanced salt tolerance in grapevine callus and cuttings, whereas silencing of this gene increased salt sensitivity. VvSR34a physically interacts with VvCOP9, and *VvCOP9* acts as a negative regulator of the salt stress response in grapevines and functions upstream of *VvSR34a*, promoting its degradation through the ubiquitin–proteasome pathway. Transcriptome analysis revealed that, under salt stress, the overexpression of *VvSR34a* leads to the upregulation of salt tolerance-related genes such as *WRKY* and *ERF*. These results provide an important target for molecular breeding of salt-tolerant grapevines, expand its application in stress-resistance genetic improvement, and lay a theoretical foundation for the grape industry to address salinization challenges.

## Data Availability

The original data for this present study are available from the corresponding authors.

## Appendix A

**Table A1:** Primers needed for the experiment.

Primer Name	Sequence (5′-3′)	Purpose
*VvSR34a*-PHBgfp-F	ggatcttccagagatATGAGTGGCCGATTTTCTCG	Construction of overexpression vector
*VvSR34a*-PHBgfp-R	ctgccgttcgacgatGGCGCTACCTGATCTGGCC	Construction of overexpression vector
*VvCOP9*-PHBgfp-F	ggatcttccagagatATGGAACCCTACTCCTTCACATCC	Construction of overexpression vector
*VvCOP9*-PHBgfp-R	ctgccgttcgacgatAGTTTCAATCATGGGTTCTGGG	Construction of overexpression vector
*VvSR34a* -F	GATCCCACCTCGTCCCCCTT	qRT-PCR
*VvSR34a* -R	AAGGGGGACGAGGTGGGATC	qRT-PCR
*VvSR34aRNAi* -F1	CGGGATCCGATCCCCCCCTCTCGTCCCCCTT	Construction of RNAi silencing vector
*VvSR34aRNAi* -R1	GCTCTAGAATGATGGTTGTGTAT	Construction of RNAi silencing vector
*VvSR34aRNAi* -F2	CGAGCTCGATCCCCCCCCCTCTCCCCCCTT	Construction of RNAi silencing vector
*VvSR34aRNAi* -R2	GGGTACCATGATGGTTGGTGTATACAAC	Construction of RNAi silencing vector
*VvCOP9RNAi*-F1	CGGGATCCGAGCTGTGATGAAGCTGAG	Construction of RNAi silencing vector
*VvCOP9RNAi*-R1	GCTCTAGAAGCTTCTCATCAGCCCT	Construction of RNAi silencing vector
*VvCOP9RNAi*-F2	CGAGCTCGAGGTGTGTGATGAAGCTGAAG	Construction of RNAi silencing vector
*VvCOP9RNAi-R2*	GGGTACCAAGCTTCTCATCAGCCCCT	Construction of RNAi silencing vector
*Vvactin*-F	GAGATTCCGTTGTCCAGAAGTC	qRT-PCR
*Vvactin*-R	CAATGTTGCCATAGAGGTCCTT	qRT-PCR
*VvSR34a*-AD-F	gccatggaggccagtgaattcATGAGTGGCCGATTTTCTCG	Yeast two-hybrid library screening
*VvSR34a*-AD-R	cagctcgagctcgatggatccGGCGCTACCTGATCTGGCC	Yeast two-hybrid library screening
*VvSR34a*-BD-F	atggccatggaggccgaattcATGAGTGGCCGATTTTCTCG	Yeast two-hybrid library screening
*VvSR34a*-BD-R	ccgctgcaggtcgacggatccGGCGCTACCTGATCTGGCC	Yeast two-hybrid library screening
PHB-GFP-R	GCATTGAACTTGACGAACGTTGTCGA	Verification of positive clones in overexpression
